# Spatial bayesian modeling of diabetes mellitus (DM) risk in the United States

**DOI:** 10.1186/s12889-025-25622-8

**Published:** 2025-11-22

**Authors:** Moses Asori, Kadae Phimia, Eric Dellmelle, Deborah SK Thomas

**Affiliations:** 1 Department of Earth, Environmental and Geographical Sciences, University of North Carolina, Charlotte, 28223 USA; 2 Department of Biostatistics and Health Data Science, Lehigh University, 27 Memorial Dr W, Bethlehem, PA 18015 USA; 3 Cartography and GIS Group, Vrije Universiteit Brussels, Pleinlaan, Brussel, 21050 Belgium

**Keywords:** Type-2 diabetes mellitus, INLA, Diabetes belt, United states

## Abstract

**Background:**

Spatial targeting of disease burden has long been instrumental to achieving optimal health resource allocation in the United States (US). Building on a previous study, the current research examined the spatial variation and hotspots of Diabetes Mellitus (DM) risk across the US.

**Methods:**

The outcome variable was the number of DM cases per county acquired from the CDC-Behavioral Risk Factor Surveillance System (BRFSS). Air pollution data (PM_2.5_ & NO_2_) were acquired from the Socioeconomic Data and Application Centre (SEDAC), obesity cases from the BRFSS, and maximum temperature data from WorldClim. The percentage of alcoholics and smokers was obtained from the County Health Rankings & Roadmaps Association. We model the spatial pattern of DM risks by coupling the integrated nested Laplace approximation (INLA) and the Local Indicator of Spatial Autocorrelation (LISA).

**Results:**

The spatial distribution of diabetes mellitus (DM) risk was non-random (I = 0.428; *p* < 0.001). The elevated risk was concentrated in the US South, where multiple counties were classified within the highest standard deviation band, at or above 2.5 standard deviations above the national expected risk (RR = 1). After adjusting for spatial confounding, smoking increased the risk of DM by 8.3% (RR = 1.083, 95% CI: 1.07, 1.095), and obesity also increased DM risk by 0.7% (RR = 1.007; 95% CI: 1.000, 1.014). However, maximum temperature (RR = 0.998; 95% CI: 0.982, 1.013), NO_2_ concentration (RR = 0.996; 95% CI: 0.984, 1.008), Social Vulnerability Index (RR = 0.99; 95% CI: 0.98, 1.001), and PM_2.5_ concentration (RR = 0.993; 95% CI: 0.97, 1.017) were not statistically significantly related to DM risk.

**Conclusion:**

The US South was found to be the region most at risk for the DM burden. Unlike previous research, our study also identified Florida and Maine as areas requiring equal attention. Although cross-sectional, the findings from this study provide essential information for targeted public health interventions.

**Clinical trial number:**

Not applicable.

**Supplementary Information:**

The online version contains supplementary material available at 10.1186/s12889-025-25622-8.

## Introduction

Diabetes mellitus (DM) is a chronic metabolic disorder characterized by elevated blood glucose levels resulting from impaired glucose secretion and insulin resistance [1]. DM is classified into two broad categories: type 1 diabetes mellitus (T1DM) and type 2 diabetes mellitus (T2DM). Type 1 diabetes mellitus (T1DM) develops due to autoimmunity against the insulin-producing beta cells of the pancreas. At the same time, T2DM is characterized by varying degrees of impaired glucose secretion, insulin resistance, and increased glucose production by the liver [1–3]. The prevalence of T1DM and T2DM is increasing worldwide, but that of T2DM is increasing at a significantly faster rate [4, 5]. DM has been linked to cardiovascular, renal, visual, and neurological complications [4]. DM is also a leading cause of adult blindness, end-stage renal disease (ESRD), and lower extremity amputation [6]. According to the International Diabetes Federation (IDF), in 2019, 463 million adults between the ages of 20 and 79 were living with diabetes, causing 4.2 million deaths worldwide [1, 7]. The IDF projects that the number of adults between 20 and 79 living with DM will likely rise to about 700 million by 2045 [1, 8]. As of 2019, 13% of Americans lived with DM, representing 34 million people, an increment of 2.5% between 2008 and 2018 [9–11]. The risk factors for DM include non-modifiable risk factors such as family history/genetic predisposition and ethnicity, and modifiable risk factors such as obesity, low physical activity, and unhealthy diet [1, 3, 9, 12]. Some studies have also linked air pollution to glucose uptake [13], hinting at its impact on DM risk. Some systematic review papers have provided evidence of an association between air pollution and DM risk, even though they highlighted the need for more studies [14, 15].

Among adults of Hispanic origin, Mexicans (14.4%) and Puerto Ricans (12.4%) had the highest prevalence of DM in the US, followed by Central/South Americans (8.3%) and Cubans (6.5%). However, among non-Hispanic Asians, Asian Indians (12.6%) and Filipinos (10.4%) had the highest prevalence, followed by Chinese (5.6%) [2]. Other Asian groups combined had a prevalence of 9.9% [10, 16]. A study found that 14% of DM cases were recorded among Whites, and about 31% were recorded among Blacks [2]. Similarly, the prevalence of smoking was 12% among Whites and 18% among Blacks [3]. The complex interaction between genetic, behavioral, socioeconomic, demographic, environmental, and metabolic factors plays a critical role in the development of DM [1, 4]. It may, therefore, complicate management efforts, including policy designs. Even though non-modifiable risk factors such as genetic factors are difficult to control, a more significant share of DM burden in the US is primarily driven by modifiable risk factors, including sedentary lifestyle, poor diet, alcohol use, smoking, as well as environmental factors such as air pollution and elevated temperature [6, 17], making them practically manageable.

The estimated diabetes-related health expenditure in the US in 2019 was $294.6 billion [18]. The mean yearly cost of a person with DM is estimated at $16,750, of which 30% is allocated directly to insulin alone [18]. The overall healthcare expenditure of an individual living with DM is estimated to be 2.3 times higher than that of people without it [18]. Therefore, DM has a significant impact on the quality of life of individuals as well as the economy. Despite substantial economic and health investments to mitigate the burden of DM in the US, it continues to have a significant impact on American residents. The inefficient realization control targets may be explained by the complexities in the interaction of underlying risk factors, further implicated by unequal health access and structural socioeconomic disadvantages [3, 19]. These environmental, socioeconomic, and behavioral risk factors, overlaid by genetic susceptibility, should be integrated to better understand their combined impacts on risk levels. Adapting existing surveillance strategies to reflect local realities is crucial for achieving spatially meaningful interventions [20, 21], including optimal resource allocation and disease screening/reporting improvements. For example, the identification of the “Stroke belt” in the mid-1960s as an area of unusual cases of stroke and associated burden [22] later helped to better target intervention and understand the etiology of stroke [19]. The spatial epidemiology of DM has received less attention. An earlier study by Barker et al. [19] acknowledged this challenge, which motivated them to initiate the process of defining the “diabetes belt.”

While the evidence provided by Barker et al. [19] needs to be updated in light of current reality, their mapping approach was also descriptive, with risk factor analysis being a spatially neutral approach [19]. This approach substantially masks our ability to decipher complex spatial processes. Following the work of Baker et al. [19], Loop et al. [3] used heat mapping to reveal the spatial distribution of DM, hypertension, and smoking across and within counties in the US. Although this study was crucial in uncovering the micro-level spatial variability of DM, it could not make an inference-based inter-county comparison of risk and driving factors, as it was also a descriptive mapping. Secondly, it was difficult to determine statistically significant spatial hotspots of DM risks at the county level. More specifically, previous studies did not address spatial variation with thorough covariate adjustment and lacked measures of uncertainty quantification. Additionally, fixed effects were not sufficiently controlled for geographic confounding, which may have led to inflated estimates of the effect size. By using fully Bayesian hierarchical disease mapping, both structured and unstructured geographical effects can be modeled, thereby addressing spatial confounding and improving accuracy. Additionally, since the diabetes hotspot was identified over a decade ago using less rigorous methods, some counties that require attention may have been overlooked, potentially reducing the effectiveness of geographic interventions. To address these gaps, our study integrates behavioral, environmental, socioeconomic, and demographic factors to map geographic distribution and identify DM risk hotspots, supporting targeted surveillance. Specifically, we rely on a coupled hotspot analysis and the Integrated Nested Laplace Approximation (INLA) – a spatial Bayesian approach- to estimate and detect hotspots of county-level risk of DM across the US. Since its inception in 2009 [23], INLA has provided a powerful modeling approach for scientists and researchers to address various health-related concerns, including managing malaria and breast cancer burdens in multiple countries worldwide [24–26]. Although the use of Bayesian techniques for spatial modeling is not new, it has relied chiefly on Markov Chain Monte Carlo (MCMC) to estimate the posterior distribution. However, due to the complicated nature of the spatial model, coupled with the large volume of data required, MCMC has been found to be computationally very expensive and more difficult to converge. INLA, therefore, offers an efficient approach for enhancing convergence and improving computational efficiency. However, to the authors’ knowledge, this approach is yet to be applied to DM spatial mapping in the US. Such an integrated and rigorous modeling framework may help update and improve the epidemiological understanding of the “diabetes belt” first identified in 2011 [19]. Specifically, our study aims [1] to map spatially varying risks of DM [2], to detect areas of statistically significant clusters of elevated risks, and [3] to identify DM risk factors.

## Materials and methods

### Data sources, description, and preprocessing

**Fig. 1:**
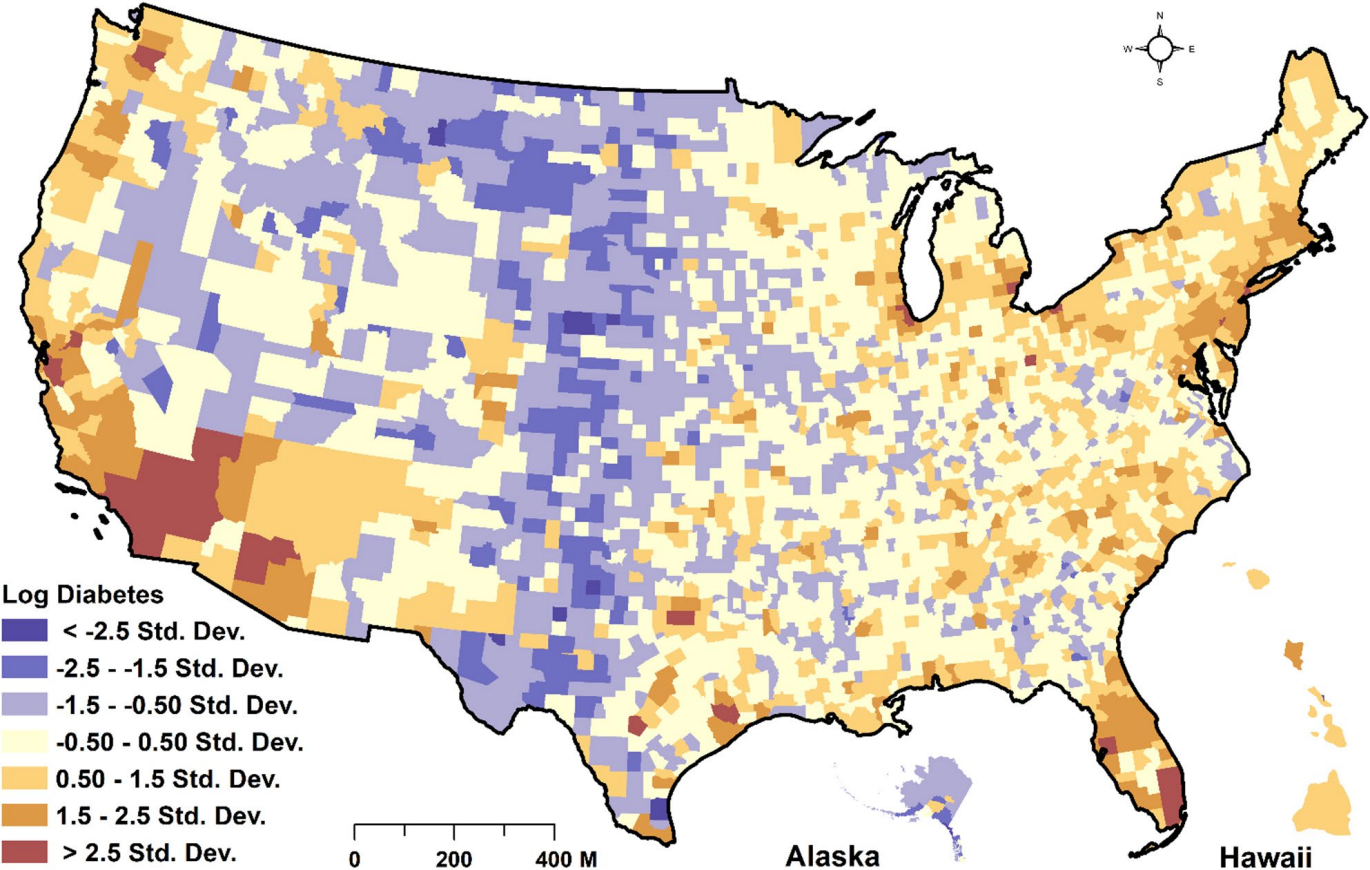
Spatial distribution of county-level SD of log count of diabetes mellitus cases

Variables to adjust for spatial effects were selected based on a literature review. For example, meta-analysis studies found that smoking increased T2DM by 37%−44% [27, 28], with bio-mechanical studies demonstrating that smoking alters the body composition, insulin sensitivity, and the pancreatic β-cell function [29]. The American Public Health Association’s review has also linked T2DM to physical activity and alcohol consumption [30]. Similarly, according to a meta-analysis of studies, those with obesity increased their risk of T2DM by about 7-fold [31]. Also, the hazard ratio for the risk of T2DM for every 2-year duration of obesity was 13% for men and 12% for women [32]. Various measures of socioeconomic status improvement also buffer against diabetes risk [33, 34]. Environmental exposures, including particulate matter (PM_2.5_) [35, 36] and NO_2_ [37, 38], have been reported to increase the risk of T2DM. Furthermore, glucose intolerance and diabetes prevalence have been found to increase with higher ambient temperature [39]. The number of DM (Fig. 1), obesity cases, and the number of physically inactive people were obtained from the US Centers for Disease Control and Prevention (CDC) active surveillance system under social determinants of health (Table 1). This data is developed from the CDC’s National Health Interview Survey (NHIS), which has been in operation since 1957, with yearly updates. Through the NHIS, data related to the health of the US population, such as disease incidence, prevalence, healthcare service utilization, and the extent of disability, are provided. A detailed description of the multistage probability design of the survey is provided in [40–42].

**Table 1:** Diabetes cases, and predictors data sources

Variables	Data year	Data Source
Diabetes cases	2023	Centers for Disease Control and Prevention (CDC); Behavioral Risk Factor Surveillance System (BRFSS) https://gis.cdc.gov/grasp/diabetes/diabetesatlas-surveillance.html#
Smoking prevalence	2023	County Health Rankings & Roadmaps https://www.countyhealthrankings.org/explore-health-rankings/alabama/data-and-resources
Obesity prevalence	2020	Centers for Disease Control and Prevention (CDC); Behavioral Risk Factor Surveillance System https://gis.cdc.gov/grasp/diabetes/diabetesatlas-surveillance.html#
PM_2.5_ concentration	2021	Socioeconomic Data and Applications Center (SEDAC) https://sedac.ciesin.columbia.edu/data/set/aqdh-pm2-5-o3-no2-concentrations-zipcode-contiguous-us-2000-2016
NO_2_ concentration	2021	Socioeconomic Data and Applications Center (SEDAC) https://sedac.ciesin.columbia.edu/data/set/aqdh-pm2-5-o3-no2-concentrations-zipcode-contiguous-us-2000-2016
Max Temperature	2020	WorldClim https://www.worldclim.org/data/worldclim21.html#google_vignette
Alcohol consumption	2023	County Health Rankings & Roadmaps https://www.countyhealthrankings.org/explore-health-rankings/alabama/data-and-resources
Social Vulnerability Index	2023	Centers for Disease Control and Prevention (CDC); Behavioral Risk Factor Surveillance System (BRFSS) https://gis.cdc.gov/grasp/diabetes/diabetesatlas-surveillance.html#

The percentage of those who engage in excessive alcohol intake and smoking was acquired from the County Health Rankings & Roadmaps data portal (Table 1). County Health Rankings & Roadmaps (CHR & R) is a program by the University of Wisconsin Population Health Institute that seeks to [1] build awareness of multiple factors that influence human health [2], provide a reliably sustainable source of local-level data to aid communities in seeing opportunities to enhance their health, and [3] bring community leaders and develop community power to enhance their health conditions [43]. For the detailed methodology of data generation and provisioning, see [43]. The CHR & R team deliberated extensively before integrating various data from several U.S. national data sources. Nitrous oxide (NO_2_) and Particulate Matter (PM_2.5_) concentrations were obtained from the Socioeconomic Data and Application Center (SEDAC) (Table 1). The number of DM and obesity cases, alcohol use, smoking, health insurance coverage, and physical inactivity were joined to polygons of 3142 counties. However, all the data above had 51 missing points (1.62%). Assuming these data points were missing at random, we imputed the missing values using multiple imputations by chained equations (mice) [44] with the random forest technique. NO_2_ and PM_2.5_ were available in raster format at 250 m resolution. Zonal statistics were used to estimate their mean values for each county across the US.

### Data and exploratory analysis

**Fig. 2:**
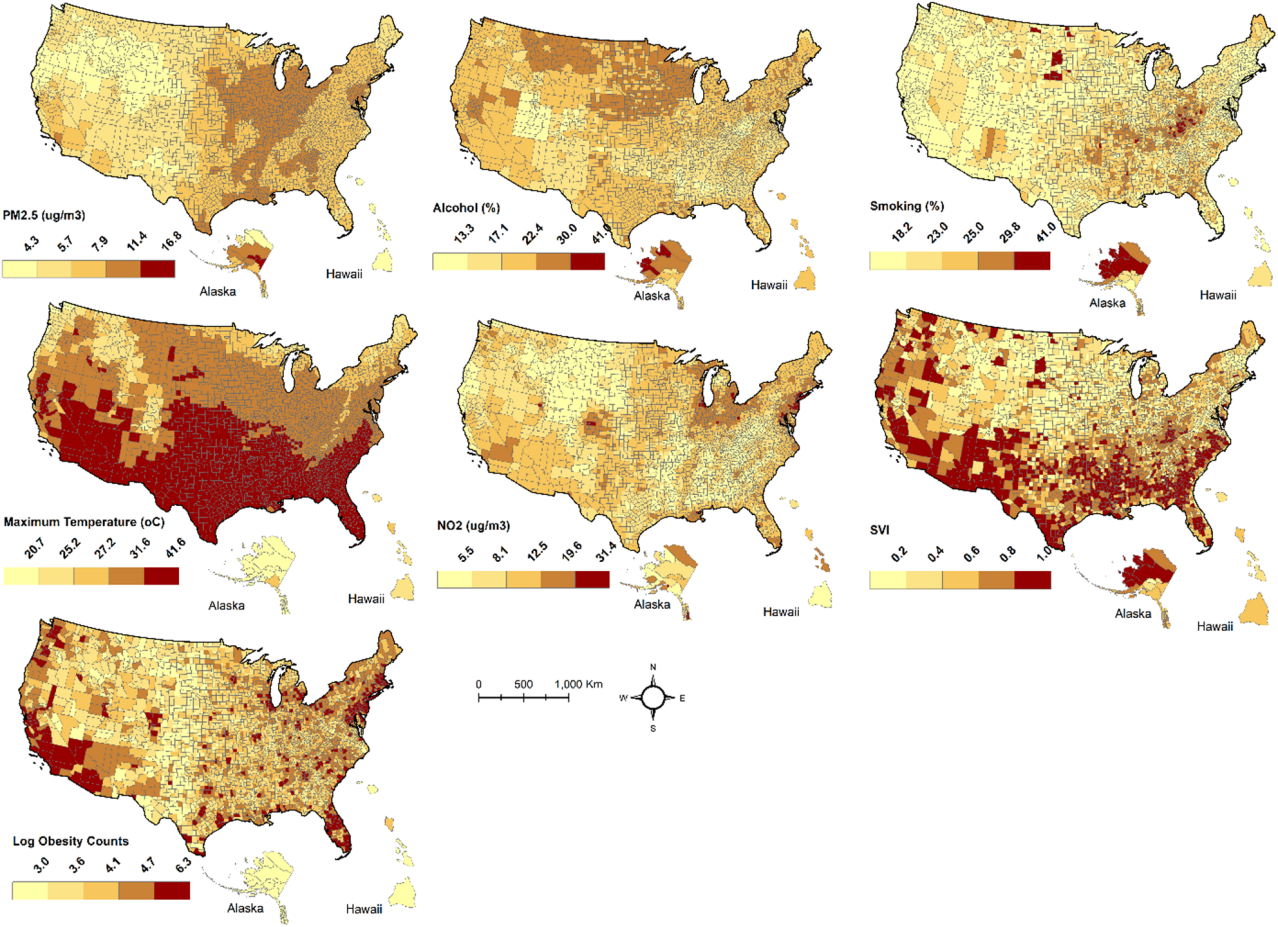
Spatial distribution of predictors. SVI represents the social vulnerability index

Our outcome variable was the total number of DM cases in each county across the US. Predictor variables included [1] the percentage of alcohol use [2], the percentage of people who smoke [3], the percentage of the population who are physically inactive [4], the percentage of the population who are obese [5], maximum summer temperature for July [6], concentration of NO_2_ [7], concentration of PM_2.5,_ [8] percentage of population with health insurance coverage [9], percentage of Black population, and [10] percentage of White population. However, to obtain a parsimonious model, we conducted a series of exploratory analyses to retain the most relevant predictors (Fig. 2). First, we computed the correlation matrix between predictor variables (supplementary file 1)*.*

Variables with *r* > 0.6 in the pairwise correlation structure were excluded. Secondly, the retained variables were entered into a stepwise-forward regression model. Variables with an adequate Bayesian Information Criterion (BIC) were included. Finally, the variable information factor (VIF) was computed. Variables with VIF > 10 were excluded from the analysis. We finally retained seven predictors that satisfied all initial scrutiny. To address potential interaction effects among the independent variables, we modeled non-spatial pairwise interactions between the seven predictors and the response variable using a negative binomial model and adjusted the *p*-values for false discovery rates (FDRs). The identified interaction terms were subsequently incorporated into the final spatial model. The spatial distribution of these predictors and the percentage of diabetes is shown in Fig. 2 below.

### Spatial and statistical analysis

Descriptive statistics were reported in means and standard errors (with 95% CI). To estimate the relative risk (RR) of DM spatially, we used INLA with the R package R-INLA [23]. The INLA model is used for spatial Bayesian inferences where neighborhood information is “borrowed” to improve the accuracy of local estimation [23–25]. Given observed counts of disease cases, the modeled count for the spatial unit $\:{Y}_{i}$ is estimated in expression one.

1 $${\mathrm Y}_{\mathrm i}\sim\mathrm P\left({\mathrm E}_{\mathrm i}\ast{\mathrm\theta}_{\mathrm i}\right),\;\mathrm i=1,2,3....,\;\mathrm n$$

Where $\:{E}_{i}$ and $\theta_i\:$ indicate the expected counts and the RR of DM for each county, respectively. Nevertheless, a reliable estimation of RR depends on essential predictors associated with DM. The RR of DM is modeled by expression two below.

2 $$\log\left(\theta_i\right)=\beta_o+\beta_1X_i+\beta_2X_2...\;...\;.\beta_nX_n+U_i+V_i$$

Where the $\:{log(\theta}_i)$ is the log of the risk coefficients, which is a linear sum of an intercept α, predictors’ fixed effects (β), and structured random ($\:{U}_{i}$), and unstructured random effects ($\:{V}_{i}$). The structured effect represents the spatial autocorrelation structure of DM cases and risk, which is information that can be used to improve our local estimation. An unstructured random effect $\:{V}_{i}$ is an unexplainable error modeled as a Gaussian noise process and assumed to be independent and identically distributed (IID). In spatial epidemiological research employing a spatial Bayesian approach, the conditional autoregressive (CAR) model and the Besag-York-Mollie (BYM) model have been widely used [22–24] to incorporate spatial autocorrelation in disease mapping and were utilized in INLA to specify the random effect model. The CAR model assumes that the expected count of disease (x) at a given location *i* is conditioned on the values of its neighbors. The CAR model is specified in expression three below as.

3 $$\:E\left({Y}_{j-i;j\ne\:i}\right)={N(\mu\:}_{i}+\rho\:{\sum\:}_{j\ne\:i}^{.}.{W}_{ij}({Y}_{i}-\mu\:)$$

Where $\:{\mu\:}_{i}$ is the expected value at county i, and *ρ* is a spatial autocorrelation parameter that determines neighboring counties’ magnitude and directional effects. The addition term is the sum of the mean adjusted values at neighboring locations *j*. However, for model selection, we tested four of them: (i) no random effects (GLM), (ii) unstructured only (IID), (iii) structured only (CAR), and BYM (combination of structured and unstructured). The Deviance Information Criterion (DIC) and the Watanabe–Akaike Information Criterion (WAIC) were then compared. Having compared these, the BYM performed better on the DIC metric and tied on WAIC with the IID, with about a 0.06 difference per county. The posterior mixing parameter was also about 0.57 (CI ≈ 0.53–0.58), suggesting the presence of both structured and instructed variation. Therefore, we chose the BYM over the others. A spatial neighborhood weight matrix was developed to define counties that were neighbors based on the Queen contiguity. Even though making Bayesian inferences has been handled with the Markov Chain Monte-Carlo (MCMC) approach, this method encounters multiple challenges, including model convergence and intensive computational requirements [23]. For details on MCMC, see Gilks et al. [45]. As a result, Harvard Rue et al. [46] developed the INLA method to address the challenges associated with MCMC. INLA is computationally less intensive and accurately approximates Bayesian inference in latent Gaussian Markov random fields (GMRF). Specifically, the model is nested in the form specified in Eqs. 4–6,

4 $$\:{Y}_{.}|X,\theta\:\:\sim\:\pi\:({y}_{i}|{x}_{i},\theta\:)$$

5 $$\:X\vert\theta\sim N\:(0,\:{Q\left(\theta\:\right)}^{-1}\:$$

6 $$\:\theta\:\:\sim\:\pi\:\:\left(\theta\:\right)$$

Where $\:{Y}_{.}$ is the likelihood, X is the Gaussian field, and $\:\theta\:$ represents the hyperparameters. The observed rates of $\:{y}_{i}$ are assumed to belong to an exponential family with mean a $\:\underset{\_}{{\mu\:}_{i}}={g}^{-1}\left({\eta\:}_{i}\right)$. The linear predictor $\:{\eta\:}_{i}$ is a linear function expressed in Eq. 7 as

7 $$\:{\eta\:}_{i}=\alpha\:+\:\sum\:_{k=1}^{n\beta\:}{\beta\:}_{k}{z}_{ki}+\sum\:_{j=1}^{nf}{f}^{\left(j\right)}\left({u}_{ji}\right)$$

The linear predictor includes various fixed, random, and unstructured effects. The structured random term $\:\sum\:_{j=1}^{nf}{f}^{\left(j\right)}\left({u}_{ji}\right)$ is defined in the modeling process to account for smooth and nonlinear functions based on specific covariates, allowing it to be more flexible and thus able to accommodate several models, including spatiotemporal models. Notably, the INLA employs both analytical estimations and numerical integration to obtain estimated posterior marginals of parameters for each GMRF component π(xi|y), i = 1…, n, based on specified priors. The joint outcome of the priors and the likelihood helps to determine the posterior distribution, which is used to quantify various parameters such as the mean and quantiles. INLA also computes the posterior marginals for hyperparameters π(θ*j*|y), j = 1…, dim(θ). Each component within the latent Gaussian field is estimated by.

 

8 $$\:{\text{x}}_{\text{i}}=\int\:{\uppi\:}\left({\text{x}}_{\text{i}}\right|{\uptheta\:},\:\text{y}\left){\uppi\:}\right({\uptheta\:}\left|\text{y}\right){\text{d}}_{{\uptheta\:}}$$

While the latent fields for the hyperparameters are estimated by Eq. 9.

 

9 $$\pi\left(\theta_j\left|y\right.\right)=\int\pi\left(\theta\left|\mathrm y\right.\right)d\theta_{-\mathrm j}$$

This nested structure is, therefore, used to calculate $\:\pi\:\left({x}_{i}\right|y)$ by coupling both the analytical estimations to the complete conditioned densities $\:\pi\:\left({x}_{i}\right|\theta\:,y)$ and $\:\pi\:\left(\theta\:\right|y)$. A numerical integration technique is used to integrate out the $\:\theta\:$. The hyperparameter’s posterior density within the latent field is particularly approximated with the Gaussian process ($\:\stackrel{\sim}{\pi\:}G\left(x\right|\theta\:,y)$. The $\:\stackrel{\sim}{\pi\:}G\left(x\right|\theta\:,y)$ is computed at the mode $\:{x}^{*}\left(\theta\:\right)={argmax}_{x}\pi\:G\left(x\right|\theta\:,y)$.

10 $$\:\stackrel{\sim}{\pi\:}\left(y\right)\propto\:\frac{\pi\:\left(x,\theta\:,y\right)}{\stackrel{\sim}{\pi\:}G\left(x,\theta\:,y\right)}|{.}_{x={x}^{*}\left(\theta\:\right)}$$

Then, INLA constructs the following nested Eqs. (12–14):

 

11 $$\:{\uppi\:}\left({\text{x}}_{\text{i}}|\text{y}\right)=\int\:\stackrel{\sim}{{\uppi\:}}\left({\text{x}}_{\text{i}}|{\uptheta\:}\right)\stackrel{\sim}{{\uppi\:}}\left({\uptheta\:}|\text{y}\right)\text{d}{\uptheta\:},\:\stackrel{\sim}{{\uppi\:}}\left({{\uptheta\:}}_{\text{j}}|\text{y}\right)=\int\:\stackrel{\sim}{{\uppi\:}}\left({\uptheta\:}|\text{y}\right)\text{d}{{\uptheta\:}}_{-\text{j}}$$

 

12 $$\:\stackrel{\sim}{\pi\:}\left(y\right)={\sum\:}_{k}^{.}.\stackrel{\sim}{\pi\:}\left({\theta\:}_{k},y\right)\stackrel{\sim}{\pi\:}\left({\theta\:}_{k}\right|y){\varDelta\:}_{k}$$

13 $$\:\pi\:\left(y\right)={\sum\:}_{I}^{.}.\pi\:\left({\theta\:}_{I}^{*}\right|y){\varDelta\:}_{I}^{*}$$

A simplified Laplace approximation, based on the Taylor expansion series, is used to accelerate computation. A fitting term (e.g., splines) may improve the approximation to the normal distribution. With the joint marginal posterior distribution of the hyperparameters, INLA searches the mode, and then, with a grid search, it finds relevant points with associated weights ($\:{\varDelta\:}_{k}$ and $\:{\varDelta\:}_{I}^{*})$ to optimize approximation to this distribution. We used a penalized complexity prior (0.01) on the marginal SD, and a moderate mixing parameter prior range (0.5, 2/3). The equality of variance with the mean was analyzed to specify the Poisson family for the INLA model. We initially fitted negative binomial and Poisson models using INLA and then compared the deviance information criteria (DIC). The overdispersion for the Poisson model was 1.26, while for the negative binomial, it was 0.74. Although the Poisson provided a moderate level of overdispersion, it had the lowest DIC (35759) compared to the negative binomial (40351). We used Poisson as the base model for our disease mapping. As a post-processing step, we assessed for spatial confounding with the predictors, a potential challenge associated with covariate-adjusted spatial modelling [47]. To test this, we compared the fixed posterior estimates with and without the structured effect. We then used an identical offset, fitted the IID and BYM models, and assessed the coefficients on the RR scale. Changes were not significant: Alcohol use 0.978→0.996 (+ 1.8%), SVI 1.010→0.990 (− 1.9%), maximum temperature 0.985→0.998 (+ 1.3%), PM_2.5_ 1.005→0.993 (− 1.2%), obesity 1.015→1.007 (− 0.8%), NO_2_ 0.987→0.996 (+ 0.8%), and smoking 1.081→1.083 (+ 0.2%). The effect sizes and signs were generally stable, suggesting limited spatial confounding. Refer to the supplementary material 2 for maps of the structured random effects. The structured random effect’s association with the predictors was modest (r = |0.29|). As a result, the spatially structured effect did not significantly proxy a single covariate. All modeling was performed in RStudio version 4.3.0 [48]. ArcMap 10.8 was then used to map the mean RR of DM. Due to the normally distributed nature of RR values, we used the standard deviation for the risk classification. Further, we conducted a local indicator for spatial autocorrelation (LISA) analysis using Anselin’s local Moran’s index to detect where statistically significant clusters of higher RR of DM occur. With LISA, four categories of clustering and outliers are reported: low-low clusters (less DM risks), high-high clusters (higher DM risks), low-high (cluster outlier), and high-low (cluster outlier). Finally, the INLA model residuals were mapped with the Getis-Ord Gi* statistic in Geoda to examine if the model adequately accounted for spatial effects.

## Results

Descriptive statistics of DM and associated predictors are found in Table 2. The average number of individuals with DM per county was 7900 (95% CI: 7117, 8775), the number of obese cases was 23,010 (95% CI: 20744, 25480), the percentage of smokers was 20.09% (95% CI: 19.945, 20.243), percentage of those who use alcohol was 19.01% (95% CI: 18.90, 19.14). Additionally, the mean PM_2.5_ concentration was 7.16 µg/m³ (95% CI: 7.105–7.219), and the NO_2_ concentration was 8.782 ppb (95% CI: 8.64–8.91).

**Table 2:** Descriptive statistics of diabetes cases and model predictors

Variables	Metric		Std. Error	95% Confidence Interval	
Alcohol use					
	Mean	19.01	0.060	18.90	19.14
	Skewness	0.342	0.044	0.194	0.536
Diabetes					
	Mean	7900.23	425.24	7117.83	8775.44
	Skewness	12.924	3.102	6.554	16.136
Obesity					
	Mean	23010.3	1229.56	20744.91	25480.47
	Skewness	12.180			
Smoking					
	Mean	20.090	0.073	19.945	20.243
	Skewness	0.218	0.076	0.078	0.384
PM25					
	Mean	7.16379	0.02962	7.10515	7.21969
	Skewness	− 0.066	0.067	− 0.179	0.076
NO2					
	Mean	8.78203	0.06923	8.64984	8.91695
	Skewness	1.390	0.091	1.215	1.580
Max Temp					
	Mean	30.42	0.0594	30.307	30.540
	Skewness	−1.132	0.111	−1.328	− 0.910
SVI					
	Mean	0.500	0.0051	0.490	0.5106
	Skewness	0.026	0.026	− 0.054	0.051

The average maximum temperature was 30.42 °C (95% CI: 30.31–30.54), and the average social vulnerability score was 0.5 (95% CI: 0.49–0.51). After adjusting for geographic effect, the posterior marginals of the fixed effects (with 95% credible intervals [CI]) showed that smoking increased the risk of DM by 8.3% (RR = 1.083, 95% CI: 1.07, 1.095), and obesity also increased DM risk by 0.7% (RR = 1.007; 95% CI: 1.000, 1.014). However, maximum temperature (RR = 0.998; 95% CI: 0.982, 1.013), NO_2_ concentration (RR = 0.996; 95% CI: 0.984, 1.008), SVI (RR = 0.99; 95% CI: 0.98, 1.001), and PM_2.5_ concentration (RR = 0.993; 95% CI: 0.97, 1.017) were not statistically significant (Table 3).

**Table 3:** The RR coefficient of the relationship between DM and predictors

Variables	RR	95% Lower Bound	95% Upper Bund	kld
Smoking behavior	1.083^a^	1.070	1.095	3.230e-11
Alcohol use	0.996	0.984	1.008	2.004e-11
Obesity	1.007^a^	1.000	1.014	2.990e-11
Social Vulnerability Index	0.99	0.980	1.001	3.177e-11
Particulate Matter (PM_2.5_)	0.993	0.970	1.017	7.377e-12
Nitrous Oxide (NO_2_)	0.996	0.984	1.008	9.834e-11
Maximum Temperature	0.998	0.982	1.013	4.009e-11

*Kld* Kullback-Leibler Divergence

^a^represents a statistically significant association

**Fig. 3:**
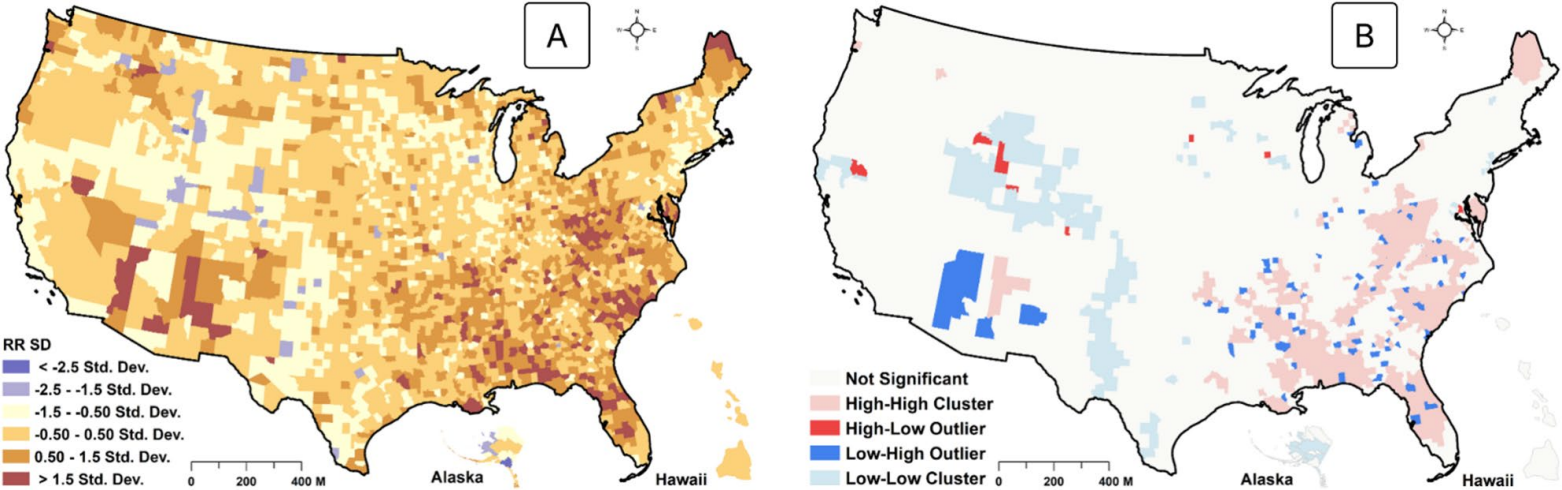
Spatial distribution of the relative risk (RR) of diabetes (county level). Map A is for the RR, and Map B is for the LISA hotspot of the RR values

The predicted cases of DM (with 95% CI) at the state level are shown in Table 4, supplementary material 1. Overall, we estimated that 24.92 million (95% CI: 22.33–27.74 million) US citizens aged 18 years and above have been diagnosed with DM (supplementary material 1). The covariates adjusted spatial distribution of the RR of DM is shown in Fig. 3 map A. Spatially, results show that the risk is more elevated in the Southeastern and western parts of the country. In particular, the very high RR category, with ≥ + 1.5 SD above expectation (RR = 1), was found in the southern US, including Miami, Florida, Mississippi, Alabama, and Louisiana, as well as in the southwestern parts, including Arizona and Nevada. Pockets of very high-risk levels were also observed in Maine, North Carolina, South Carolina, and Virginia (Fig. 3 map A). RR within + 0.5 and + 1.5 SD were observed in similar spatial patterns as the very high-risk category. Medium risk levels of −0.50 to + 0.50 SD were sporadically distributed nationwide. However, the states of Nevada, Utah, Wyoming, and Arkansas recorded between − 1.5 and − 2.5 SD below the national expected RR of 1.0, indicating significantly lesser risk.

**Fig. 4:**
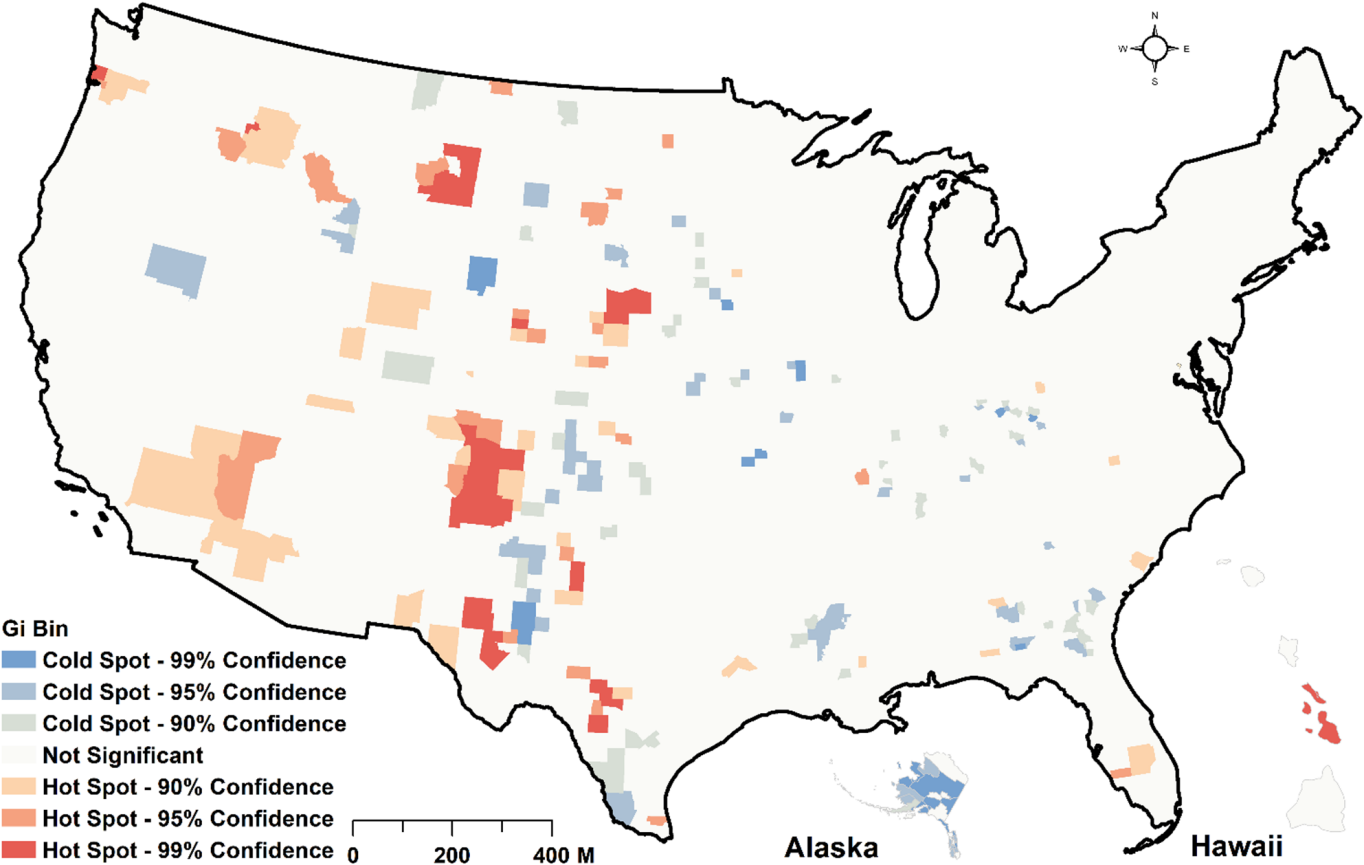
A hotspot analysis of model residuals

As shown in Fig. 3 maps A and B below, four general patterns of spatial clusters of DM risk zones can be visually observed, with the majority of these patterns observed in the South. Specifically, a statistically significant hotspot (high-high) of the RR of DM was detected in Maine and the Southern US. However, pockets of cluster outliers were detected for some counties in Nevada, where low-risk counties were surrounded by high-risk ones (low-high clusters). We also observed statistically significant lower-risk zones (low-low groups) in the Midwest and Southwestern parts of the country (Fig. 3 map B). Fewer risk zones were also detected in areas around Texas. In Alaska, low-risk clusters were detected. No statistically significant clusters were detected in Hawaii. We also mapped model residuals to detect the autocorrelation structure of model errors. The global Moran *I* (I =−0.00131; *p* = 0.1234) indicated no spatial autocorrelation in model residuals. As also shown in Fig. 4, almost all locations exhibited no spatial autocorrelation for the error terms. Therefore, the model was generally adequate in handling the substantive portion of the spatial random effects.

## Discussion

Spatial targeting efforts have long been instrumental in achieving optimal public health resource allocation in the US, with the “stroke belt” concept being a notable example [21]. Like obesity and stroke, studying the spatial nature of the “diabetes belt” is crucial to its management [20]. The prevalence of DM has been increasing since 1999 due to the combined impacts of multiple factors, including the Patient Protection and Affordable Care Act, which improved screening and survival rates [49]. Besides, the increased prevalence of obesity, smoking, and socioeconomic disadvantages has also added to the existing DM burden, thus intensifying the need for more targetable intervention [6, 19]. Taking these together, our study extends prior research conducted by Baker et al. [19] to provide an updated picture of the “diabetes belt” discourse. Principally, the pattern detected by our study is similar to the “diabetes belt” delineated by Baker et al. in 2011 [19]. Counties in the Southern US (except a large part of North Carolina), Maine, and Nevada had a + 1.5 SD of RR of DM above the reference (Fig. 2). As expected, areas within the Midwest had lower RR than other parts of the country, with most counties having approximately − 1.5 and <−2.5 SD RR of DM below the reference level (Fig. 3 map A). Of course, this observation was not surprising, as counties in the US South have a long history of profound social, economic, and healthcare-related inequalities and increased environmental injustices [50–52].

Smoking and obesity were statistically significantly associated with an increased risk of DM. Given a causal association, smoking increased the risk of type 2 DM by 44% [27]. Similarly, among 41,810 men recruited for the Health Professional Follow-up study, the RR of type 2 DM was 1.94 among men who smoked 25 or more cigarettes daily compared with nonsmokers [53], corroborating our current finding. It is, therefore, not surprising that a higher prevalence of smoking in the US South was associated with an increased risk of DM after adjusting for relevant spatial confounders. Obesity significantly strengthens the linkage between diabetes and health function and health perception in the US [54]. In our study, alcohol consumption was inversely linked to DM risk at the population level, but it was not statistically significant. Additionally, we did not find a statistically significant marginal association between NO_2_, PM_2.5,_ maximum temperature, and DM after accounting for geographic confounding. This finding differs from the results of Dian He et al.‘s meta-analysis on the impact of PM_2.5_ on T2DM risk [55]. This difference may be attributed to variations in study designs, as the meta-analysis included only aspatial cohort studies, which may not account for spatial confounding effects. Secondly, while we used cross-sectional data for our spatial model, the meta-analysis relied on cohort studies that captured long-term exposure. Our result should, therefore, be interpreted with caution, especially when compared with longitudinal, Cohort, and non-spatial studies since our model parameters are spatially adjusted. Despite this, this study provides valuable information for county and state-level targeting efforts. Policymakers may have to target al.l these risk factors that increase the risk of DM and counties where these combined effects are substantial. To the authors’ knowledge, this is the first study to apply a critical lens to the US “diabetes belt,” first identified by Baker et al. [19] in 2011. However, some differences require highlighting. The “diabetes belt” delineated by Baker et al. [19] was based on quantile breaks of the percentage of diagnosed DM cases, in which counties ≥ 11% were designated as diabetes risk zones. Nevertheless, ours is more extensive, where an RR epidemiologic measure was estimated, with 1.5 SD above the reference risk level (RR = 1) designated as tier 1 risk zones. In addition, we assessed whether clusters of these at-risk zones were statistically significant, a spatial detail that had not been previously considered.

Following this distinction, it is apparent that the earlier study underestimated the spatial extent and number of the counties at risk of diabetes. For example, our study identified counties in Florida and Maine as high-risk zones for DM. In contrast, they were excluded from the “diabetes belt” delineated by Baker et al. [20]. Secondly, some counties in Nevada and New Mexico were identified as risk zones in our study (Fig. 3 map A). Nevertheless, these areas were not part of the “diabetes belt” in the previous study. Increased prevalence of DM in Florida has been attributed to increased disparities in the distribution of factors such as obesity/overweight, socioeconomic status (SES), and the existence of comorbidities such as hypertension and hypercholesterolemia over the years [56–58]. It is also important to note that the newly discovered counties may be due to temporal factors. For example, diabetes prevalence has been increasing since 1999 because of changing lifestyles, environmental exposures, and increased resources for more screening. Given this new evidence, policymakers and practitioners may need to update their decision-making information to include Maine, Florida, and counties in Southern Nevada and New Mexico. Beyond identifying more counties for prioritization, the RR estimates in our studies have been geographically adjusted for behavioral, socioeconomic, environmental, and spatial effects, making them reliable for identifying target areas. Additionally, by classifying RR levels based on SD from the reference value (RR = 1), we support a tiered intervention framework. For example, counties with an SD greater than 1.5 are assigned to tier 1 for intensive programs. Those with SD between 0.5 and 1.5 fall under tier 2 for outreach programs, while counties with SD between − 0.5 and 0.5 are placed in tier 3 for regular surveillance.

While our study is rigorous enough to uncover spatial patterns of at-risk counties, several limitations are worth noting. Behavioral Risk Factor Surveillance System data may be subject to recall bias, social desirability bias, and the inability to reach households without landline telephones [19]. However, our study used nationwide data, thus increasing its statistical power to detect spatially meaningful details. Secondly, at the county level, spatial variation may be masked. Therefore, studies at a finer geographic scale (e.g., census tract level) will further strengthen public health interventions. Thirdly, our data relied on only diagnosed DM cases. Therefore, adding undiagnosed cases may increase the extent and number of counties designated as risk zones in our study, as most undiagnosed cases are likely to be located within the US South, where the combined effects of social, economic, and healthcare disadvantages are pervasive. We also provided a cross-sectional risk picture based on 2023 DM cases. Although this is updated data, it does not indicate whether the spatial pattern remains temporally consistent. Spatiotemporal INLA modeling or spatiotemporal satScan statistical modeling may help verify the temporal consistency of DM hotspots across the US.

## Conclusion

The research assessed the spatial variability and hotspots of DM risk across the US. Findings are consistent with previous studies regarding risk factor analysis and the spatial distribution of DM risk, although newer counties have been detected and added to the “at-risk counties”. Specifically, elevated risk levels of DM were detected primarily in the Southern US, where socio-economic and healthcare-related disparities are prevalent. Therefore, targeting these counties that are already overlain by multiple challenges may be crucial. Even though our risk modeling integrated environmental, socioeconomic, and behavioral risk factors, the posterior marginals of fixed effects showed that only obesity and smoking were statistically significantly related to DM risk after adjusting for geographic confounding. Integrating the combined effects of these complexities with spatial random effects, our model effectively reveals more apparent patterns of counties at an elevated risk of DM. For example, counties previously undetected were observed in our current model. Therefore, public health policymakers and practitioners may update their list of “at-risk counties’ to include counties in Florida, Maine, Southern Nevada, and New Mexico, which were previously excluded. We also estimated the posterior counts of DM cases at the state level, which could be helpful information for state-level decision-making processes.

## Supplementary Information

Supplementary Material 1

Supplementary Material 2

## Data Availability

The dataset(s) supporting the conclusions of this article are included within the article (see Table 1).

## Glossary

SEDAC: Socioeconomic Data and Application Centre
INLA: Integrated nested Laplace approximation
LISA: Local Indicator for Spatial Autocorrelation
T2DM: Type 2 diabetes mellitus
BRFSS: Behavioral Risk Factor Surveillance System
CDC: Centers for Disease Control
