# Supplementary material for: Spatial bayesian modeling of diabetes mellitus (DM) risk in the United States
Source: BMC Public Health. 2025 Nov 22;25:4422. doi: 10.1186/s12889-025-25622-8 (PMC12754990; doi:10.1186/s12889-025-25622-8)
Supplement: Supplementary file 1 — Supplementary Material 1 [file 12889_2025_25622_MOESM1_ESM.docx]

Table 1: RR difference between IID and BYM estimates. RR_1 is for IID, and RR_2 is for BYM. Pct =percentage.

| predictor | RR_mean_1 | RR_LCL_1 | RR_UCL_1 | RR_mean_2 | RR_LCL_2 | RR_UCL_2 | delta_RR_pct | delta_beta_sd |
| --- | --- | --- | --- | --- | --- | --- | --- | --- |
| Alcohol | 0.978236 | 0.97085 | 0.985681 | 0.996174 | 0.984385 | 1.008108 | 1.833657 | 4.701082 |
| Svi | 1.009559 | 1.000511 | 1.018689 | 0.990297 | 0.980048 | 1.000651 | -1.90796 | -4.19675 |
| Temp Max | 0.984516 | 0.977293 | 0.991793 | 0.997513 | 0.982471 | 1.012813 | 1.320137 | 3.493116 |
| Pm25 | 1.005146 | 0.997587 | 1.012763 | 0.993024 | 0.969746 | 1.016854 | -1.20598 | -3.15226 |
| Obesity | 1.015199 | 1.007997 | 1.022453 | 1.006732 | 0.999587 | 1.013926 | -0.83404 | -2.30714 |
| No2 | 0.987314 | 0.979635 | 0.995052 | 0.99556 | 0.98364 | 1.007666 | 0.835161 | 2.089247 |
| Smoking | 1.080646 | 1.071174 | 1.090198 | 1.082587 | 1.070382 | 1.094925 | 0.179623 | 0.399898 |

Table 2: The pairwise interaction coefficients between the predictors. P_fdr is the false discovery rate adjusted p-values

| term | IRR | LCL | UCL | beta | se | z | p | delta_AIC | p_fdr |
| --- | --- | --- | --- | --- | --- | --- | --- | --- | --- |
| Smokingxobesity_int | 1.04 | 1.032 | 1.049 | 0.039 | 0.004 | 9.219 | 0 | -79.595 | 0 |
| smokingxsvi_int | 0.988 | 0.981 | 0.994 | -0.013 | 0.003 | -3.59 | 0 | -10.313 | 0.001 |
| svixpm25_int | 1.008 | 1.001 | 1.015 | 0.008 | 0.003 | 2.351 | 0.019 | -3.412 | 0.037 |
| svixno2_int | 1.006 | 0.999 | 1.013 | 0.006 | 0.004 | 1.724 | 0.085 | -0.955 | 0.127 |
| tempxobesity_int | 1.004 | 0.999 | 1.009 | 0.004 | 0.003 | 1.463 | 0.144 | 0.096 | 0.156 |
| pm25xmax_temp_int | 1.006 | 0.998 | 1.014 | 0.006 | 0.004 | 1.418 | 0.156 | 0.018 | 0.156 |

Table 3: Model selection measures.

| model | DIC | WAIC |
| --- | --- | --- |
| BYM2 | 35758.85618 | 35106.26138 |
| CAR only | 35769.27954 | 35127.961 |
| IID only | 35814.91736 | 35142.26303 |
| GLM (no spatial) | 169393.4334 | 10386245.25 |


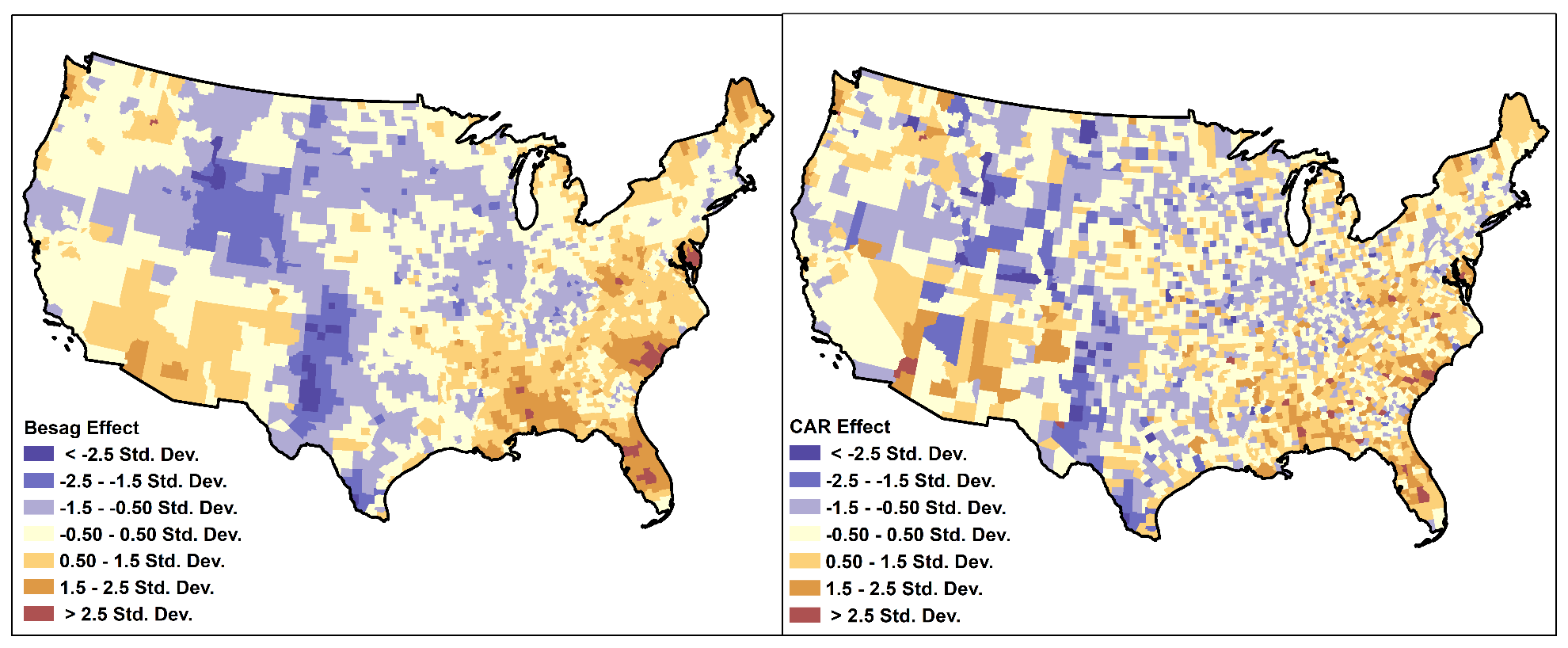


Figure 1: Distribution of structured random effect estimates for Besag and CAR models.


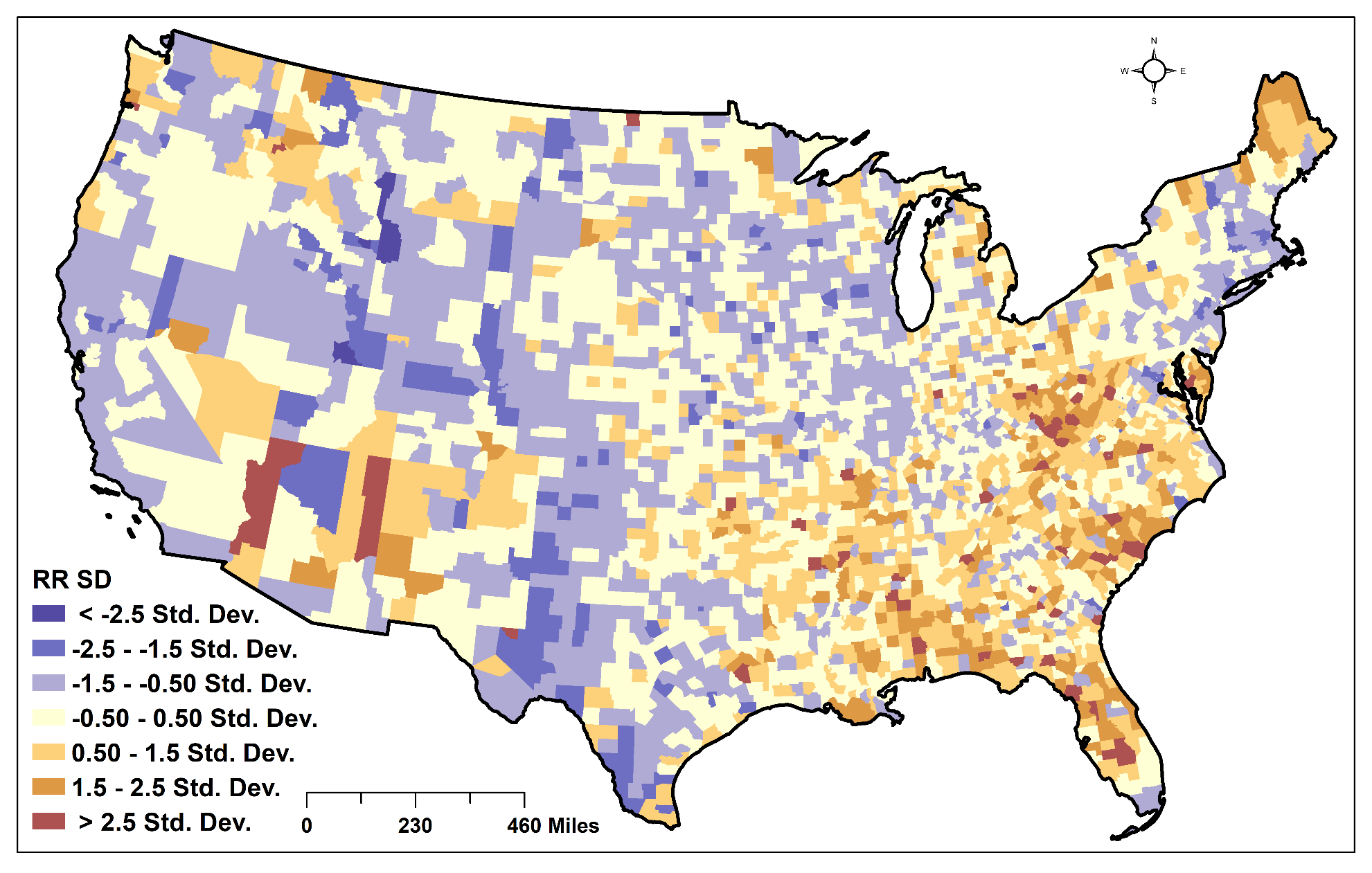


Figure 2: RR distribution based on unadjusted interaction effects.
