# Supplementary material for: Spatial bayesian modeling of diabetes mellitus (DM) risk in the United States
Source: BMC Public Health. 2025 Nov 22;25:4422. doi: 10.1186/s12889-025-25622-8 (PMC12754990; doi:10.1186/s12889-025-25622-8)
Supplement: Supplementary file 2 — Supplementary Material 2 [file 12889_2025_25622_MOESM2_ESM.docx]

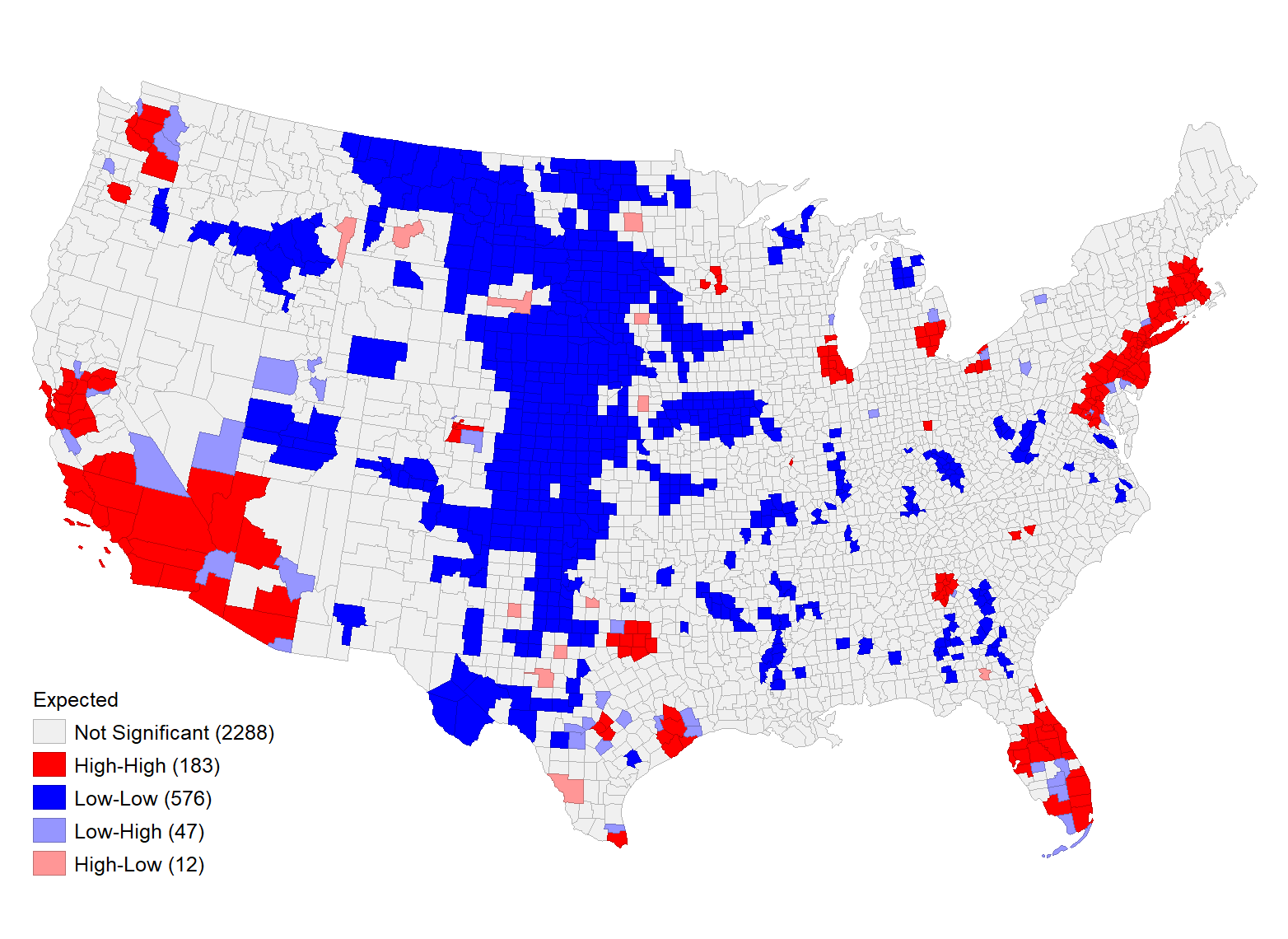


Fig 1: LISA map for the expected number of Diabetes cases


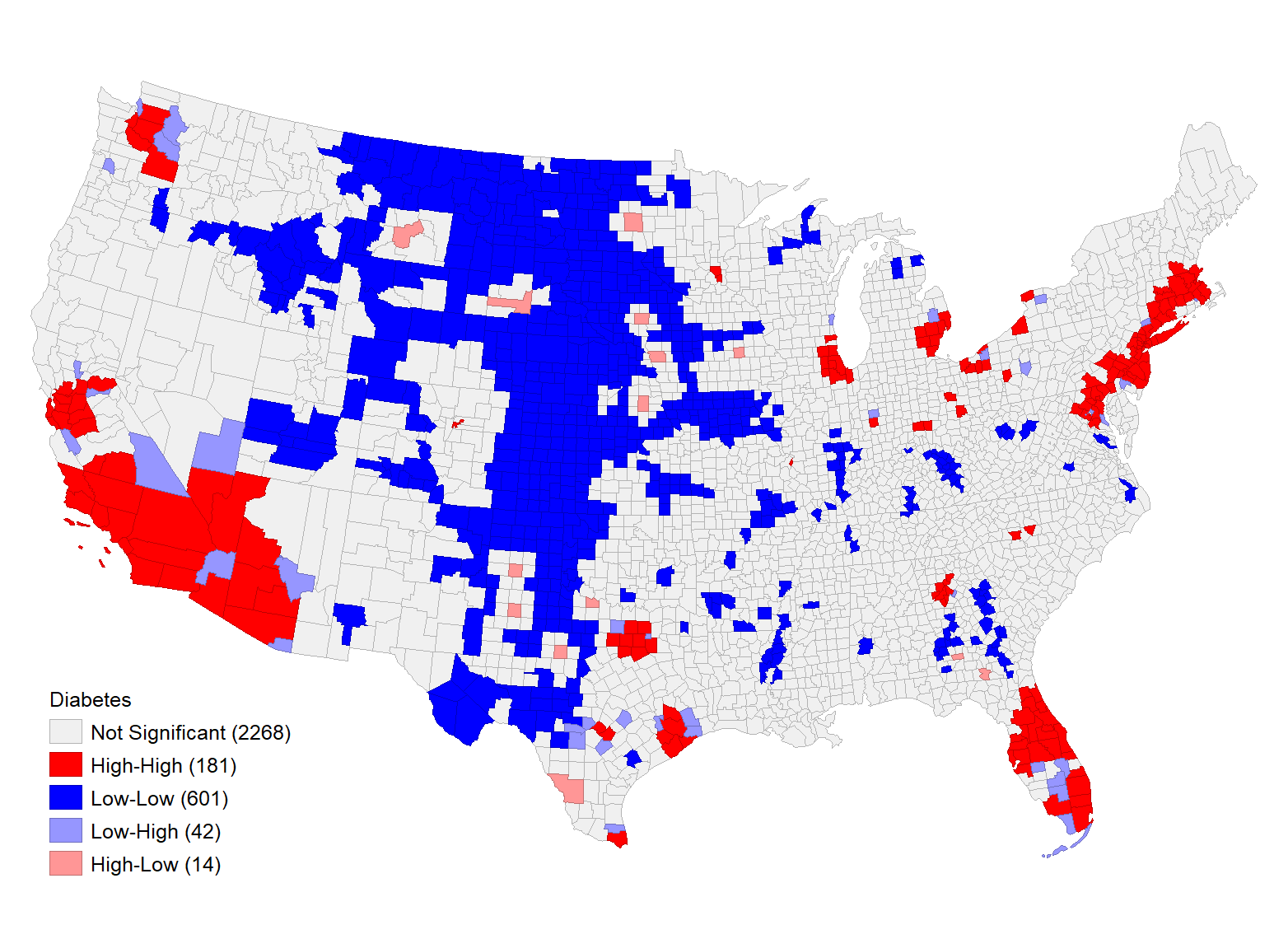


Fig 2: LISA map for observed cases of Diabetes.


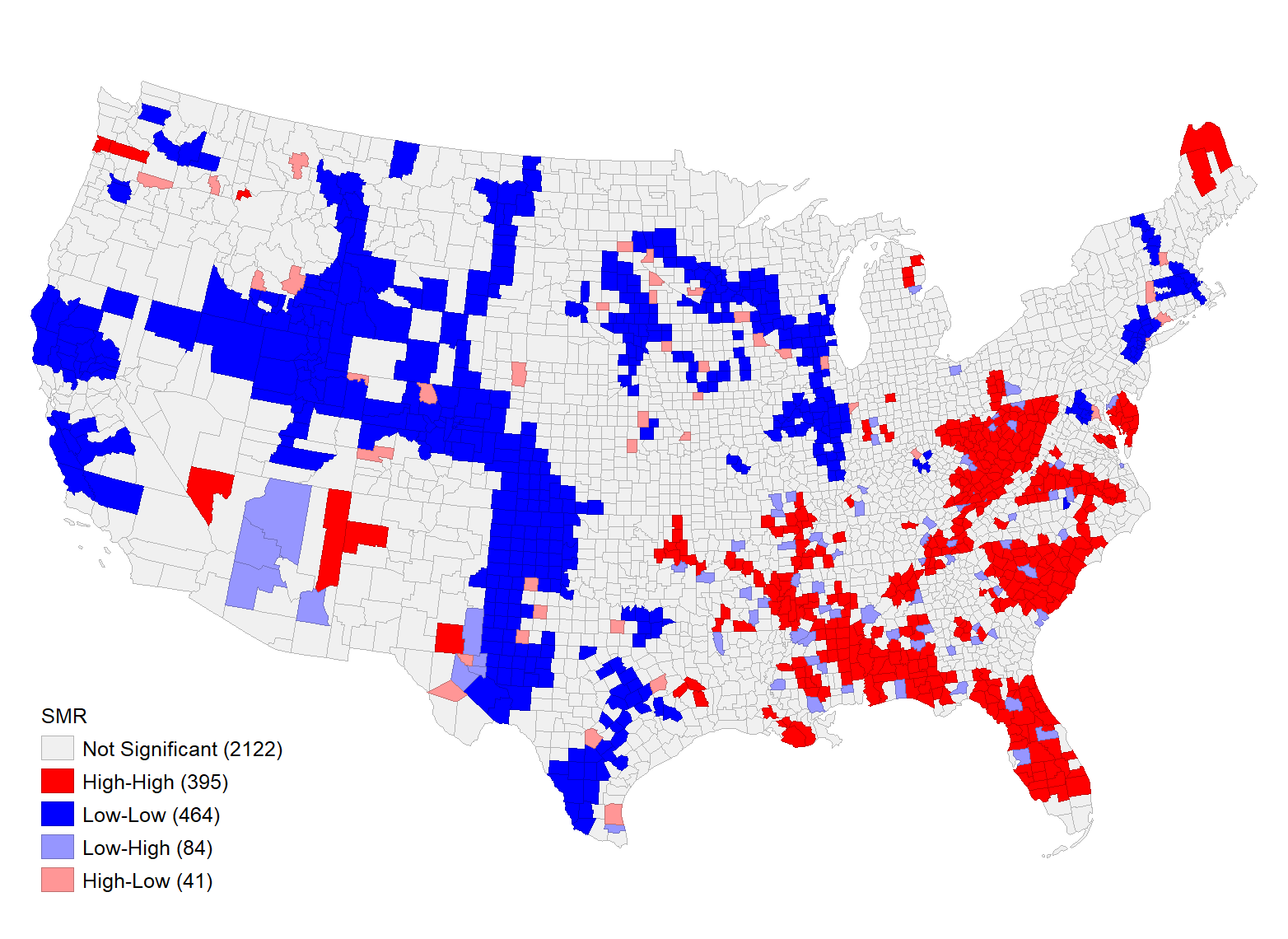


Fig 3: LISA map for SMR


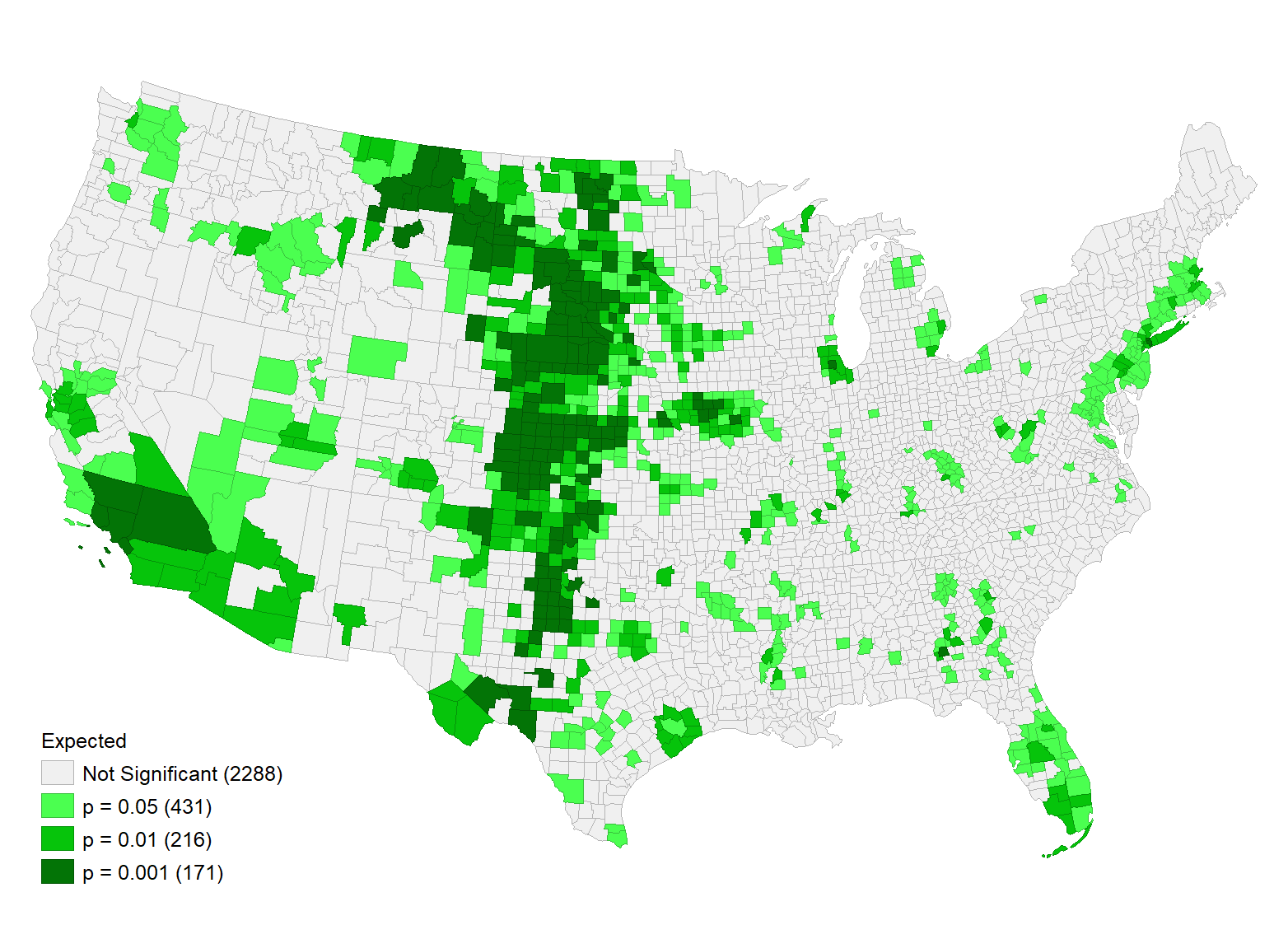


Fig 4: P-value for LISA for expected cases of diabetes


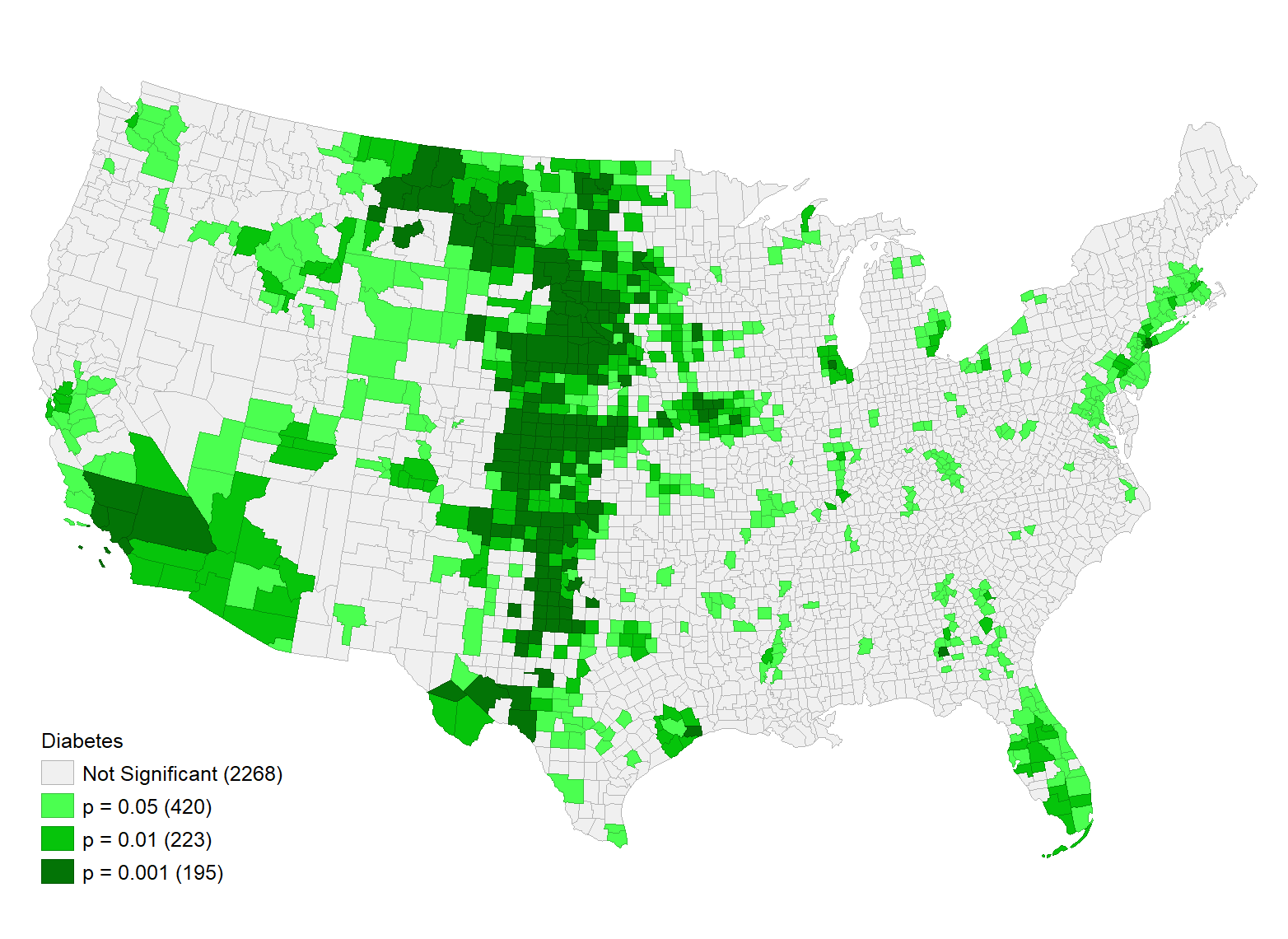


Fig 5: P-value for Diabetes cases clustering


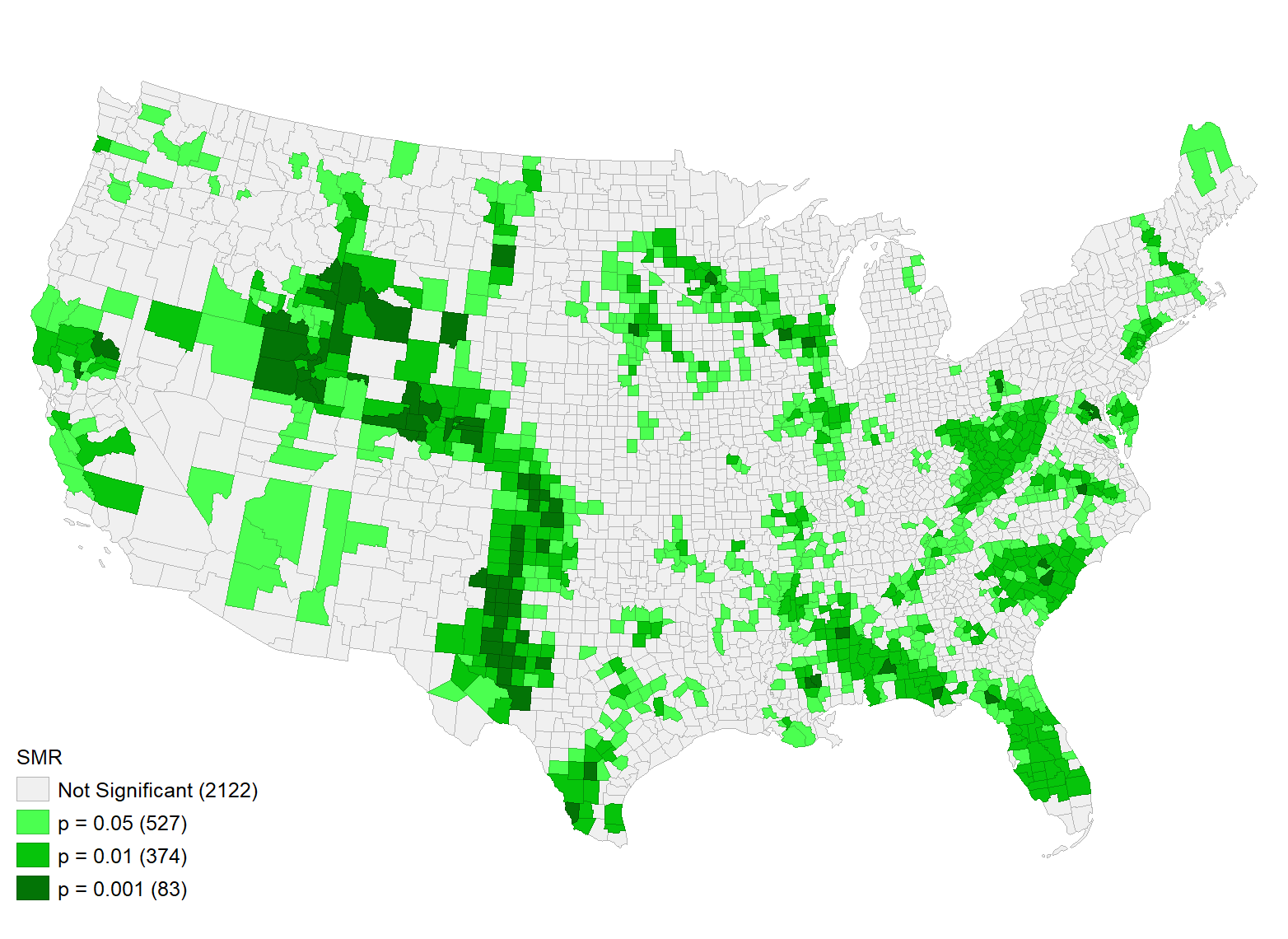


Fig 6: P-value for diabetes SMR clustering


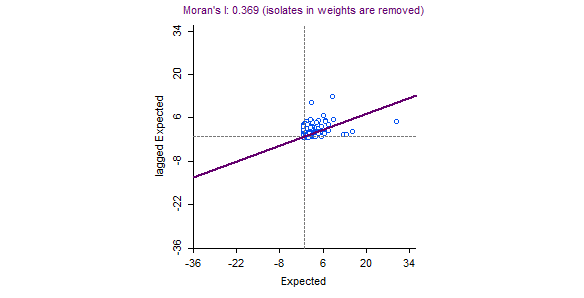


Fig 7 Moran, I scatter plot for expected cases of diabetes


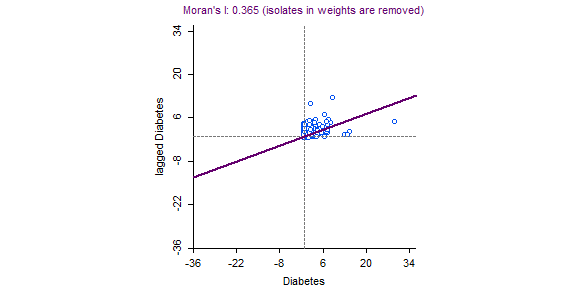


Fig 8; Moran’s I for observed cases of diabetes
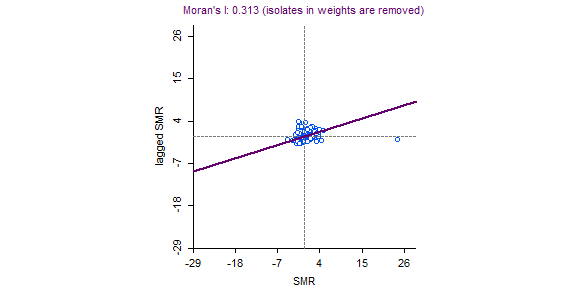


Fig 9: Moran’s I for SMR of diabetes


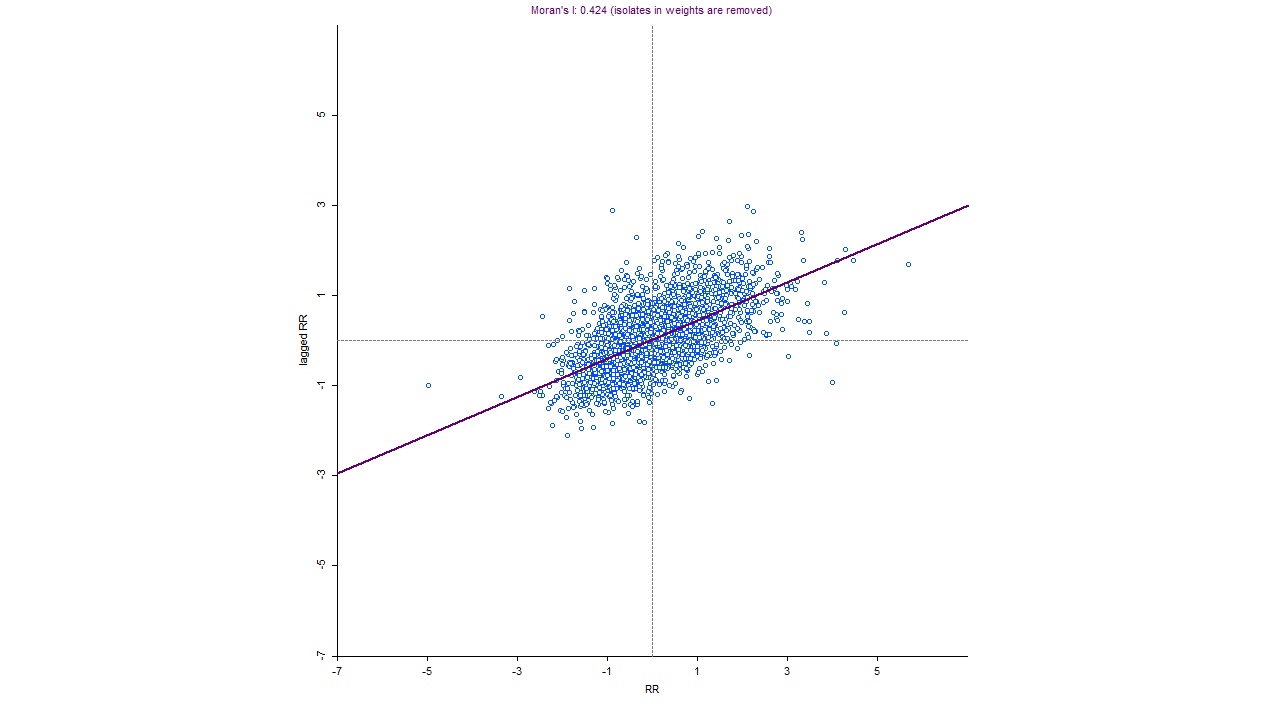


Fig 10: Moran’s scatter plot for RR of diabetes


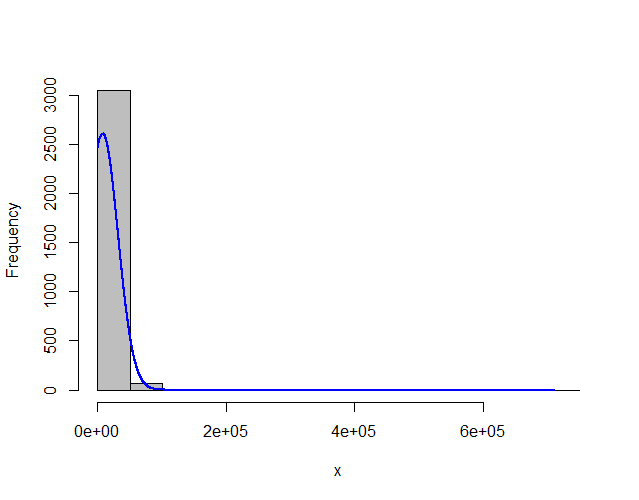


Fig 10: Histogram of observed diabetes cases


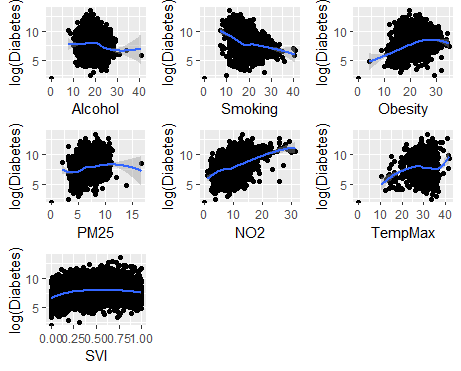


Fig 11: Bivariate association between diabetes and predictors


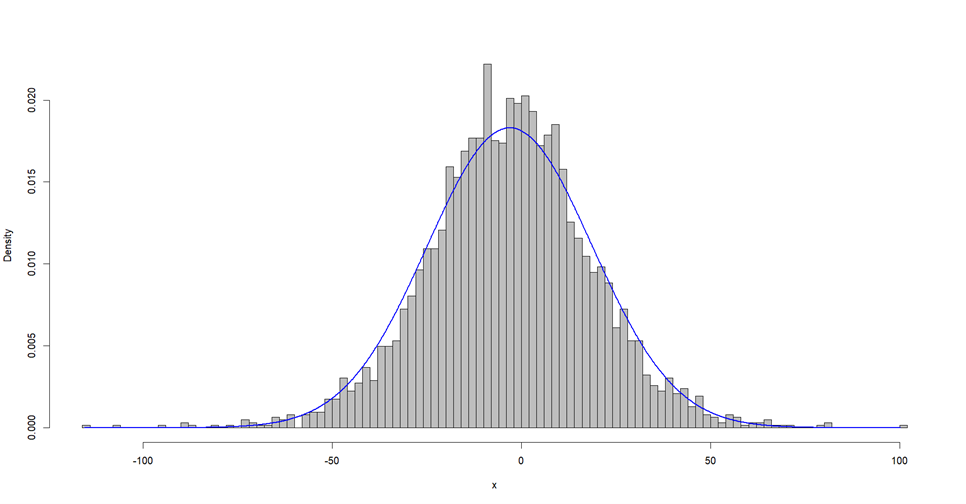


Fig 12: INLA-based model residuals of diabetes cases


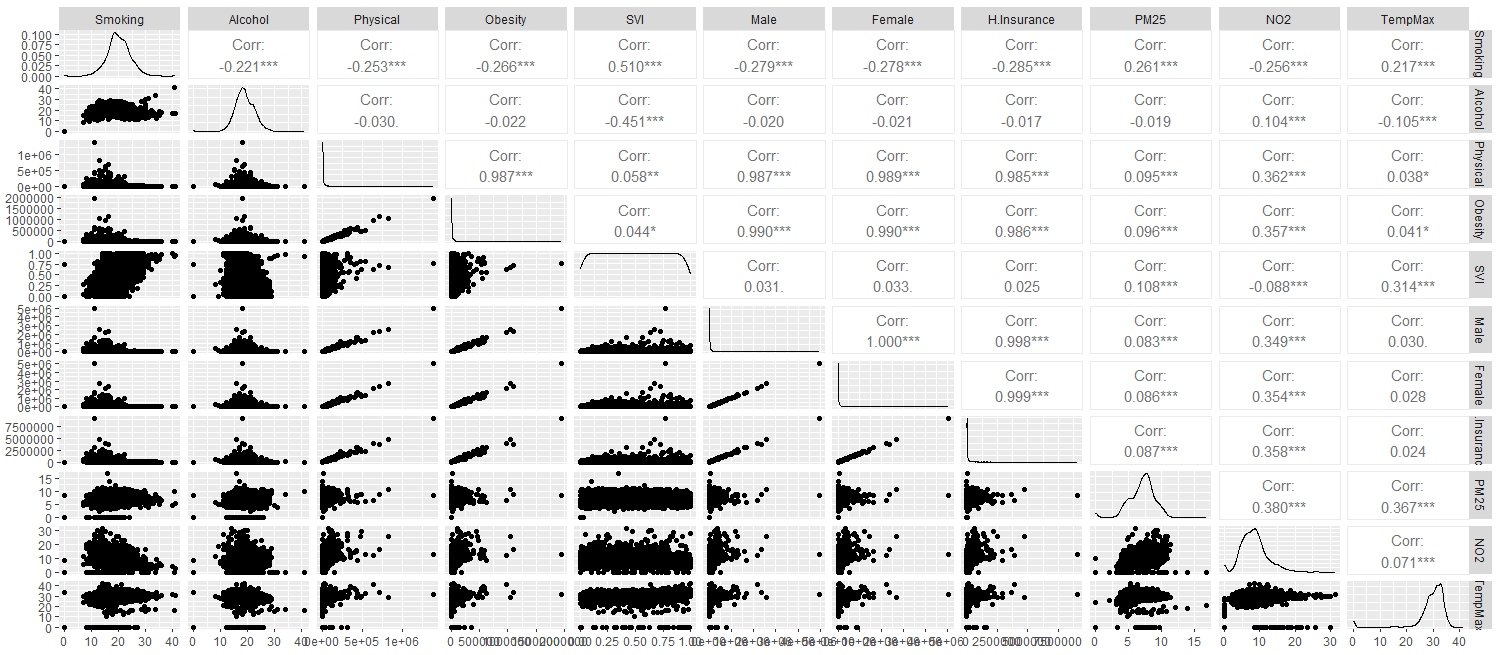


Fig 14: Correlation matrix

Table 4: Estimated cases of DM in the state with 95% credible intervals.

| States | Estimate | 95% LL | 95% UL |
| --- | --- | --- | --- |
| Alabama | 454585 | 410131.2 | 502599.9 |
| Alaska | 48778.45 | 43155.1 | 54859.88 |
| Arizo0 | 567589.4 | 509004.8 | 631234.1 |
| Arkansas | 260004 | 234293.4 | 287764.4 |
| California | 2709477 | 2407545 | 3039428 |
| Colorado | 350605.2 | 315557.7 | 388173 |
| Connecticut | 247626.4 | 220953.1 | 276561.7 |
| Delaware | 89331.42 | 79917.59 | 99579.31 |
| Florida | 1894023 | 1679708 | 2128260 |
| Georgia | 851403 | 767552.5 | 941625.5 |
| Hawaii | 104088.5 | 89716.58 | 120204.6 |
| Idaho | 128184.7 | 114772.1 | 142666.2 |
| Illinois | 847015.1 | 761577.8 | 939770.9 |
| Indiana | 547297.8 | 493957.7 | 604950.6 |
| Iowa | 232549 | 210466.6 | 256232.5 |
| Kansas | 221149.1 | 199696.1 | 244239.3 |
| Kentucky | 356563.2 | 321852.5 | 393851.2 |
| Louisia0 | 399111.4 | 358122.5 | 443524.6 |
| Maine | 110288 | 97819.36 | 123901.6 |
| Maryland | 461251.6 | 411709.4 | 515328.8 |
| Massachusetts | 445986.1 | 395827 | 500719.6 |
| Michigan | 801033.7 | 715092 | 894453.3 |
| Minnesota | 380987.6 | 343326.9 | 421365.5 |
| Mississippi | 276946.1 | 249336.7 | 306770.9 |
| Missouri | 470348.4 | 423991.8 | 520259.1 |
| Monta0 | 77679.66 | 69944.83 | 86023.9 |
| Nebraska | 142160.9 | 128064.9 | 157346.5 |
| Nevada | 247685.3 | 223659.8 | 273834.6 |
| New Hampshire | 100693.5 | 90467.75 | 111714.2 |
| New Jersey | 620301.7 | 554430.6 | 692060.6 |
| New Mexico | 160454.6 | 144034.5 | 178229.5 |
| New York | 1409444 | 1247830 | 1587164 |
| North Carolina | 867142.8 | 781099.4 | 959562.4 |
| North Dakota | 56979.91 | 51224.16 | 63162.38 |
| Ohio | 977175.6 | 881198.9 | 1080768 |
| Oklahoma | 323424.7 | 291834.7 | 357431.7 |
| Oregon | 307848.7 | 276143.5 | 342068 |
| Pennsylvania | 997483.3 | 899620.6 | 1102867 |
| Rhode Island | 79846.73 | 71357.05 | 89081.97 |
| South Carolina | 483757.7 | 435855.7 | 535721.9 |
| South Dakota | 62204.34 | 56120.42 | 68724.89 |
| Tennessee | 582183 | 526533.2 | 642058.1 |
| Texas | 2108670 | 1894573 | 2339650 |
| Utah | 185754.2 | 166844.8 | 205986.1 |
| Vermont | 42949.49 | 38423.68 | 47824.61 |
| Virginia | 665239.1 | 595828.2 | 740462.2 |
| Washington | 540733.4 | 483482 | 602623.2 |
| West Virginia | 181900 | 164024.5 | 201264.7 |
| Wisconsin | 406400.5 | 364145.9 | 452066 |
| Wyoming | 40424.46 | 36445.7 | 44654.26 |
| Total | 24924761 | 22328272 | 27740676 |

UL=95% upper limit; LL=95% lower limit.
